# Command Disaggregation Attack and Mitigation in Industrial Internet of Things

**DOI:** 10.3390/s17102408

**Published:** 2017-10-21

**Authors:** Peng Xun, Pei-Dong Zhu, Yi-Fan Hu, Peng-Shuai Cui, Yan Zhang

**Affiliations:** 1 Department of Electronic Information and Electrical Engineering, Changsha University, Changsha 410022, China; 2 College of Computer, National University of Defense Technology, Changsha 410073, China; xunpeng12@nudt.edu.cn (P.X.); ouchyf@foxmail.com (Y.-F.H.); cuipengshuai@nudt.edu.cn (P.-S.C.); 3 Department of Informatics, University of Oslo, Oslo 0316, Norway; yanzhang@ieee.org

**Keywords:** cyber-physical attack, industrial Internet of Things, command disaggregation, command correlation, attack detection

## Abstract

A cyber-physical attack in the industrial Internet of Things can cause severe damage to physical system. In this paper, we focus on the command disaggregation attack, wherein attackers modify disaggregated commands by intruding command aggregators like programmable logic controllers, and then maliciously manipulate the physical process. It is necessary to investigate these attacks, analyze their impact on the physical process, and seek effective detection mechanisms. We depict two different types of command disaggregation attack modes: (1) the command sequence is disordered and (2) disaggregated sub-commands are allocated to wrong actuators. We describe three attack models to implement these modes with going undetected by existing detection methods. A novel and effective framework is provided to detect command disaggregation attacks. The framework utilizes the correlations among two-tier command sequences, including commands from the output of central controller and sub-commands from the input of actuators, to detect attacks before disruptions occur. We have designed components of the framework and explain how to mine and use these correlations to detect attacks. We present two case studies to validate different levels of impact from various attack models and the effectiveness of the detection framework. Finally, we discuss how to enhance the detection framework.

## 1. Introduction

A large-scale industrial Internet of Things (IIoT) [1] is deployed to help utilities such as smart train and smart grid provide better service. A typical hierarchical system is adopted in many large-scale IIoTs to obtain flexible control [2,3,4]; this system includes many lower-layer sub-controllers, such as programmable logic controllers (PLCs) in power systems. Sub-controllers are in charge of command disaggregation. For example, a demand response (DR) load reduction of 70 MW in power grid is requested across the entire grid. The command needs to be disaggregated into some sub-commands such as load reduction of 10 MW, 20 MW, and 40 MW according to the capacity of endpoint field devices because the appliances have different levels of capacity. This disaggregation process continues until local commands for endpoint field devices are generated and exercised [5,6].

However, with the wide openness of communication infrastructure which is used to improve efficiency, reliability, and sustainability of services [7] such as smart grid, new vulnerabilities have been exposed [8,9,10,11,12]. High-skilled attackers can obtain many opportunities to remotely access sub-controllers to inject malicious commands and modify data from sensors. A real case was studied in [13] to demonstrate this ability of smart attackers.

When commands reach sub-controllers, malicious entities remotely attack sub-controllers to generate wrong executed commands called **command disaggregation attack**. The attack may result in disruptions of physical process. In this paper, we focus on the process of launching the command disaggregation attack and its detection method. Previous studies, such as [2], introduced the concept of command disaggregation attack. Attackers can inject false commands or modify sensory data to implement false command disaggregation. However, these studies did not describe how to launch effective command disaggregation attacks to result in damages to the physical system. Besides, when attackers simultaneously launch command disaggregation attacks and inject false feedback data to confuse the security detector, existing detection methods such as false data estimation [14] can not effectively identify anomalies. Detecting command disaggregation attacks with false feedback data injection is still an unexplored topic.

Driven by the above considerations, we depict two different command disaggregation attack modes: (1) false command sequence; and (2) wrong command allocation. The former refers to the situation that attackers delay the disaggregation of some commands to disorder its logic, thereby resulting in disruptions of physical process; the latter refers to the situation that disaggregated commands are issued to other than the expected or planned actuators, causing the failure of control objective or physical damages. We also describe three attack models to implement command disaggregation attacks in two kinds of modes. When attackers manipulate the disaggregation of commands, they simultaneously inject false feedback data to confuse security detectors to ensure that the attack goes undetected. To deal with the threats above, we provide a detection framework based on correlations among two-tier command sequences, which collects two-tier commands including those issued from the central controller and the sub-commands executed by actuators. We design components of the detection framework and explain the method of mining correlations among commands and using the correlations to detect attacks. Finally, two cases are studied to demonstrate the different levels of impact from various attack models and the effectiveness of proposed detection framework.

The rest of the paper is organized as follows. We introduce the related work and summarize our contributions in Section 2. We depict two kinds of modes and three attack models in Section 3. In Section 4, we describe the detection framework. Two case studies are given in Section 5. We discuss how to enhance the detection framework in Section 6 and conclude our work in Section 7.

## 2. Related Work and Our Contribution

### 2.1. Related Work

In this section, we first survey the state of the art of attacks that cause disruptions of physical process. Then, we review the works about attack detection.

Three methods, namely, false command injection, false data injection, and time-delay attacks, can be used to disrupt the physical system. In [15,16,17], attackers directly injected false commands into the controller to disturb physical process. False data injection attack was described in [18,19]. Attackers capture sensors to inject false data, which causes false state estimation or hides signs of faults leading to disruptions. In [7,20], authors described time-delay switch attack, which increases time delays in the sensing loop or in the automatic generation control signal to impact the control process. In [21], authors considered DoS attacks. Attackers jammed communication channels by intruding into the advanced metering infrastructure of smart grid. Commands and feedback data can not be transmitted to the actuators and controller thereby compromising the control process. In [13], authors described a real case that attackers can manipulate sub-controllers by infecting the firmware of a PLC. An attacker gets access to the PLC’s input values through the firmware from the physical world, processes them, and then provides outputs that are forwarded to the physical world through the firmware. Moreover, attackers also can modify feedback data which are transmitted to the central controller from the compromised PLC. These studies showed that the existing vulnerabilities in IIoTs can enable attackers to remotely disrupt physical process, but they did not consider the command disaggregation attack. Currently, there are some researches that have focused on command disaggregation attacks. For example, in [2], the authors demonstrated the possibility of command disaggregation attacks and revealed the cascading failure effect, but the process of command disaggregation attacks that can cause damage to physical system is not described.

Although many detection methods have been proposed to detect anomalies caused by attacks, they are not effective to identify false command disaggregation attacks. For example, in [22], some counter-attack mechanisms were proposed to defend attacks, however, attackers may find vulnerabilities and bypass these mechanisms to launch false command disaggregation attacks by injecting elaborately constructed false data or directly intruding into the controllers [2]. In [23,24], authors used the linear correlations among sensory data to detect anomalies. However, when false command disaggregation occurs and attackers simultaneously inject false data to confuse the security detectors [14], anomalies can not be detected. Likewise, false data estimation that used multiple data detectors with dissipativity-theoretic fault detection function in [14] and detection method based on correlations between commands and sensory data in [25,26] also failed in identifying the above attacks. In [27], authors used methods based on machine learning to detect attacks, however, when bad data is successively injected, the method is still ineffective to identify command disaggregation attacks. In [28,29], authors mined the correlations among commands to detect injected false commands. However, command disaggregation attacks modify commands after commands are collected from the controller, which leads to the undetected situation. If defenders only collect commands from actuators, multi-variant types of commands will increase the difficulty and inaccuracy of correlation mining.

### 2.2. Our Contribution

After summarizing the related work in attack and in detection, we clarify our contributions as

(i) We introduce two kinds of command disaggregation attack modes, namely, false command sequence and wrong command allocation.
(ii) We describe three attack models to implement command disaggregation attacks in two modes. Attacks based on the three models can not be detected by the existing detection methods.
(iii) We provide an effective detection framework based on correlations among two-tier command sequences. Detecting command disaggregation attacks with false feedback data injection is still an unexplored topic and our method is the first to effectively identify command disaggregation attacks before a disruption occurs.

## 3. Command Disaggregation Attack

In this section, we first introduce a simplified model of IIoT control system. Second, we unveil two kinds of command disaggregation attack modes, including wrong command allocation and false command sequence, wherein we depict attack models.

### 3.1. System Model

**Figure 1:**
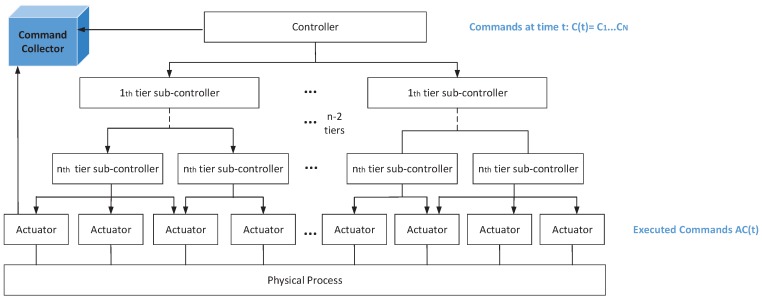
The structure of IIoT control system.

Figure 1 shows the structure of a typical IIoT control system, which is composed of the central controller, sub-controllers, actuators, and sensors. The central controller issues command sequences based on the physical system state and sends these commands to the corresponding sub-controllers. Sub-controllers are responsible for command disaggregation and feedback data transmission from sensors to the central controller. There exist multi-tier sub-controllers. The sub-controller in the upper level sends disaggregated commands to the sub-controllers in the lower tier. Sub-commands are gradually disaggregated until the sub-controllers in the lowest level send sub-commands to the actuators. Actuators execute sub-commands to implement the physical process and the physical system state has a change. The current physical system state is evaluated based on values of sensors, and then new commands are issued to further control the physical process. An example of the command disaggregation is shown in Figure 1, where commands C(t)=$\left\{c_{1},\ldots ,c_{n}\right\}$ are issued simultaneously from the central controller at time *t*. After multi-tier sub-controllers disaggregate these commands, sub-commands AC(t) are executed by the actuators. $c_{i}$ denotes a kind of command and AC(t) denotes the executed sub-commands, which are defined in subsequent description of the system model.

The system model is described using six-tuple:

(1) P={C,Ts,S,AC,Re,Fs}

where

- $C=\{c_{1},\ldots ,c_{m}\}$ is a finite set of commands from the central controller. $c_{k}$ is the *k*th kind of command. $C\left(t\right)=\left\{c_{i},\ldots ,c_{j}\right\}$ indicates the commands issued by the central controller at time *t*.
- $Ts=\{ts_{1},\ldots ,ts_{nd}\}$ is a finite set of time series. A time series is the measured values of one sensor with the change of time. $ts_{i}={\left\{ts_{i}\left(1\right),\ldots ,ts_{i}\left(k\right)\right\}}^{T}$ means the time series from the *i*th sensor. $ts_{i}\left(l\right)$ denotes the measurement of the *i*th sensor at time instant *l*. nd means the number of sensors.
- $S=\{s_{1},\ldots ,s_{n}\}$ is a finite set of physical system states. $s_{j}={\left\{a_{1},\ldots ,a_{nd}\right\}}^{T}$ denotes the *j*th kind of state and $a_{i}\in R$. Detectors and controllers can evaluate the system state at time *k*, S(k), based on values of sensors, which can be computed by
  (2) $$\bar{S(k)}=C_{matrix}\times Ts\left(k\right)$$
  where $C_{matrix}\in R^{nd\times nd}$ is the constant matrix. $\bar{S(k)}\in S$ denotes the evaluated state at time *k*, and under normal circumstances, $\bar{S(k)}=S\left(k\right)$. Ts(k) = ${\left\{ts_{1}\left(k\right),\ldots ,ts_{nd}\left(k\right)\right\}}^{T}$ where $ts_{i}\left(k\right)$ denotes the value of time series $ts_{i}$ at time instant *k*.
- $AC=\{AC_{11},\ldots ,AC_{ij},\ldots ,AC_{mn}\}$ is a set of sub-commands executed by actuators. $AC_{ij}=\;{\left\{ac_{ij}\left(1\right),\ldots ,ac_{ij}\left(N\right)\right\}}^{T}$ indicates the executed sub-commands by actuators when command from the central controller is $c_{i}$ and the system state is $s_{j}$. Element $ac_{ij}\left(k\right)$ defines the sub-command that will be executed by the *k*th actuator. *N* means the number of actuators. An actuator only executes a sub-command in unit time, and a sub-controller only disaggregates one command from the upper-tier sub-controller during once outflow of the central controller. $AC\left(t\right)={\left\{ac_{i_{1}j_{1}}\left(1\right),\ldots ,ac_{i_{N}j_{N}}\left(N\right)\right\}}^{T}$ denotes the executed sub-commands when the corresponding commands C(t) are issued from the central controller. AC(i,t) is an element of AC(t) and denotes the sub-command executed by the *i*th actuator. The system state at time *t*, S(t), is decided by $\bar{S(t-d_{t})}$ and $AC(t-d_{t})$ [14], which can be described as
  (3) $$S\left(t\right)=A\times \bar{S(t-d_{t})}+B\times AC\left(t-d_{t}\right)$$
  where $A\in R^{nd\times nd}$ and $B\in R^{nd\times N}$ are constant matrices. $d_{t}$ indicates the time interval between the time *t* when current commands are issued and the time of its last outflow.
- $Re=\{r_{1},\ldots ,r_{m\times n}\}$ is a finite set of relationships among commands and system states. $r_{d}=<\;s_{j},c_{i},AC_{ij}>$ ($r_{d}\in Re$) indicates that the executed sub-commands are $AC_{ij}$ when the system state is $s_{j}$ and the command from the controller is $c_{i}$.
- $Fs=\{fs_{1},\ldots ,fs_{y}\}$ is a subset of set *S*. A disruption occurs when the system state is $fs_{i}$.

The model is based on the assumption that the information and physical systems have not yet been attacked, and all observed states and commands can be regarded as a representation of normal system behavior. From the above process, we can know that the accurate feedback data and commands are critical for the normal running of systems. When security mechanisms such as authentication [30] and cryptography [31] are used to protect the data from sensors to controllers and commands from the controller to the sub-controllers, false command injection and bad data injection can be launched with less possibility. However, when attackers control the PLC’s firmware below the control logic by compromising a device through the Joint Test Action Group interface [13], these mechanisms may become ineffective. Besides that, adding authentication and cryptography mechanisms may be unwelcome in many existing IIoTs because of a large amount of investment. Moreover, security mechanisms may delay the response from the physical system, which can not be accepted by some real-time systems. In follow-up studies, we assume that attackers can bypass these mechanisms or focus on vulnerable systems without these security mechanisms.

We also use $subCom(c_{i})$ to denote a set. Any element $x\in subCom(c_{i})$ satisfies

$$\left\{\begin{array}{c} x\in AC\\ <c_{i},s_{j},x>\in Re \end{array}\right.$$

where $s_{j}$ is any possible system state when command $c_{i}$ is issued.

To describe the attack models, we define two operations about sets, “−” and “+”. For any two sets $Q_{1}$ and $Q_{2}$, $Q_{1}+Q_{2}=\left\{e|e\in Q_{1}\cup e\in Q_{2}\right\}$ and $Q_{1}-Q_{2}=\left\{e|e\in Q_{1}\cap e\notin Q_{2}\right\}$.

### 3.2. Two Kinds of Attack Modes and the Attack Models

In this section, we will disclose two kinds of attack modes and describe the corresponding attack models in details. During the implementation of the attack models, attackers usually inject false data into sensors or feed back false data to detectors to hide signs of attacks.

#### 3.2.1. Wrong Command Allocation

**Figure 2:**
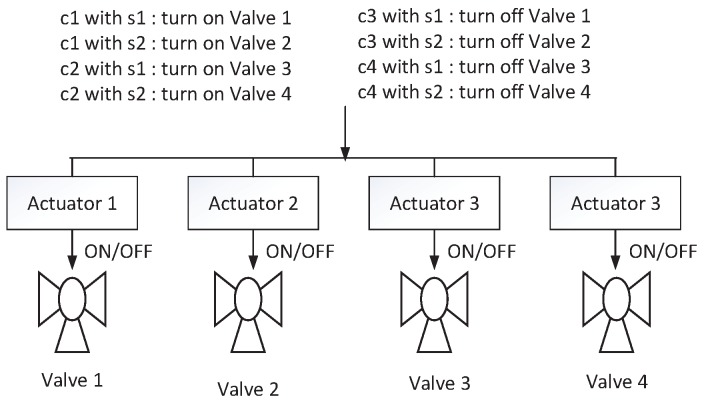
An example: explaining different attack modes.

When a command $c_{i}$ is disaggregated at system state $s_{k}$, the sub-commands may be sent to false actuators or changed to other sub-commands leading to a situation that sub-commands $AC_{ik}$ are changed to $AC_{jl}$. There exist two situations about wrong command allocation mode, including wrong command inner allocation and wrong command outer allocation. Wrong command inner allocation occurs when an attacker changes the executed sub-commands $AC_{ik}$ to the false sub-commands $AC_{jl}\in subCom\left(c_{i}\right)$. Wrong command outer allocation occurs when attackers change $AC_{ik}$ to false sub-commands $AC_{jl}\notin subCom\left(c_{i}\right)$. In Figure 2, an example is shown to explain the two situations. c1,c2,c3,c4 are commands from the controller, and s1,s2 are physical system states. Commands at different system states can turn on/off valves. When the current command is $c_{1}$ and the system state is $s_{1}$, if attacks make Valve 2 turned on, this situation is called wrong command inner allocation. If attacks turn Valves 3 or 4 off or on, this situation is called wrong command outer allocation.

Next, we depict the attack models that implement the above two situations.

(1) Attack model based on wrong command inner allocation (WCIA)

**Figure 3:**
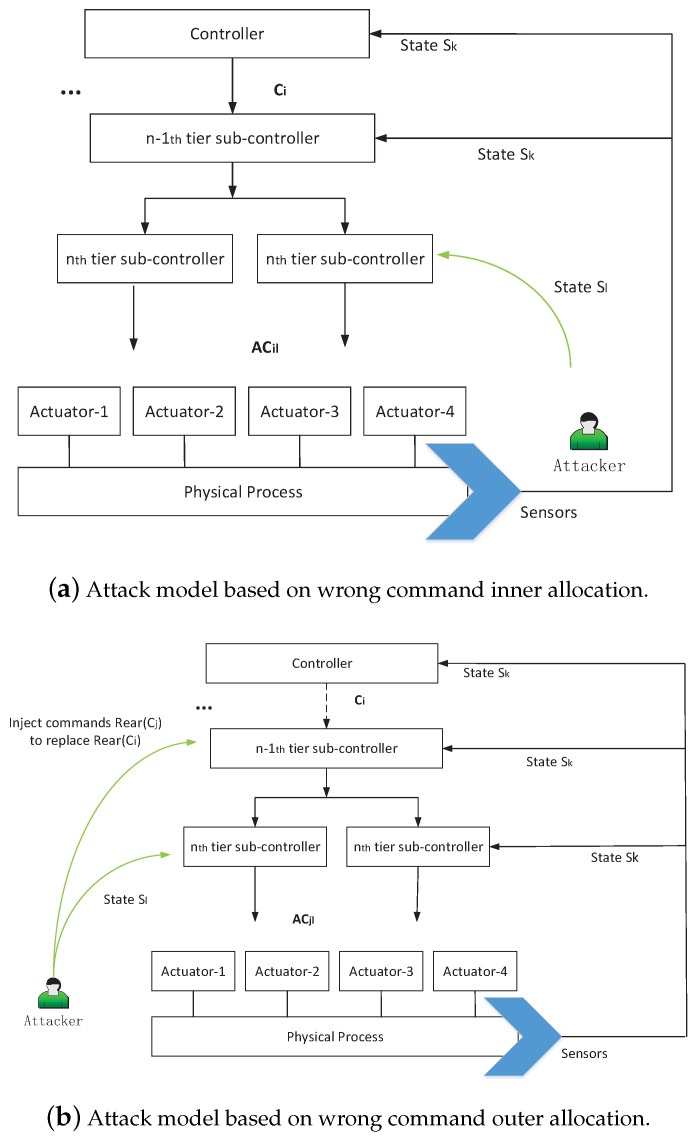
Attack models based on wrong command allocation mode.

$AC_{jl}\in subCom\left(c_{i}\right)$ means j=i. To achieve this target, attackers can inject false data to interfere with the state estimation. As shown in Figure 3a, WCIA is described as follows:

• Information collection

Attackers first find a set of issued commands C(t), command $c_{i}$, state $s_{k}$, and state $s_{l}$ satisfying Equation (4).

(4) $$\left\{\begin{array}{c} c_{i}\in C\left(t\right),\\ \;<s_{k},c_{i},AC_{ik}>\in Re,\\ \;<s_{l},c_{i},AC_{il}>\in Re,\\ s_{m}=A\times s_{k}+B\times \left(AC\left(t\right)-AC_{ik}+AC_{il}\right),\\ s_{m}\in Fs. \end{array}\right.$$

• False data injection

When attackers discover that the current state is $s_{k}$ and command $c_{i}$ will be disaggregated, attackers inject bad feedback data into sub-controllers in charge of the disaggregation of $c_{i}$ and inform them the current state is $s_{l}$. Attackers need to control different levels of sub-controllers based on different demands. If attackers inject false feedback data into the *i*th sub-controllers, they also need to inject the same false feedback data to manipulate the corresponding (*i* + 1)th tier sub-controllers. The disaggregation of commands is influenced and the executed sub-commands are changed from $AC_{ik}$ to $AC_{il}$. A disruption occurs when $AC_{il}$ are executed because the system state becomes $s_{m}\in Fs$. To go undetected as described before, attackers again inject false feedback data after $AC_{il}$ was executed, which needs to change the state from $s_{m}$ to $s_{i}$ ($s_{i}=A\times s_{k}+B\times AC\left(t\right)$). Unlike the former false feedback data injection, this false state should be obtained by the central controller and sub-controllers, which means that attackers should directly inject false data into sensors or feed back the false state to all controllers.

(2) Attack model based on wrong command outer allocation (WCOA)

$AC_{jl}\notin subCom\left(c_{i}\right)$ means j≠i. To achieve the target, attackers not only need to inject bad feedback data, but also to modify the command, as shown in Figure 3b. WCOA is described as follows:

• Information collection

Attackers first find a set of issued commands C(t), commands $c_{i}$ and $c_{j}$, state $s_{k}$, and state $s_{l}$ satisfying Equation (5).

(5) $$\left\{\begin{array}{c} c_{i}\in C\left(t\right),\\ \;<s_{k},c_{i},AC_{ik}>\in Re,\\ \;<s_{l},c_{j},AC_{jl}>\in Re,\\ s_{m}=A\times s_{k}+B\times \left(AC\left(t\right)-AC_{ik}+AC_{jl}\right),\\ s_{m}\in Fs. \end{array}\right.$$

• Command modification

We use $Rear(c_{i})$ to denote the disaggregated commands of $c_{i}$ by the middle-tiers sub-controllers. Unlike the wrong command inner allocation, attackers first change the commands $Rear(c_{i})$ to $Rear(c_{j})$ when $c_{i}$ has been disaggregated as $Rear(c_{i})$, and then transfer them to the next-tier sub-controllers.

• False data injection

When disaggregated commands have been modified, attackers need to inject bad feedback data to the next-tier sub-controllers. Bad data informs the next-tier sub-controllers that the current state is $s_{l}$. The real situation is $s_{k}$. If the commands received by the next-tier sub-controllers still require disaggregation, attackers should inject false feedback data to its next-tier controllers. Thus, attackers should try to control the nearest sub-controllers from the actuators to decrease the number of compromised sub-controllers.

When the sub-commands $AC_{jl}$ were executed by actuators, attackers should also re-inject false feedback data to confuse the controller. The state should be changed to $s_{i}$ ($s_{i}=A\times s_{k}+B\times AC\left(t\right)$).

#### 3.2.2. False Command Sequence

Under normal situations, if $c_{i}$ is executed before $c_{j}$ is issued from the central controller, then actuators should first execute sub-commands from the disaggregation of $c_{i}$. Sub-commands from the disaggregation of $c_{j}$ then are executed. From the controller’s point of view, $<c_{i},c_{j}>$ stands as sequential commands, however, the actuators execute the sequence $<c_{j},c_{i}>$ when attacks based on false command sequence occur. To achieve the target, attackers need to delay the disaggregation of command $c_{i}$, meanwhile, inform the controller that $c_{i}$ has been executed and command $c_{j}$ should be issued from the controller. After $c_{j}$ is executed, $c_{i}$ is disaggregated. For example, in Figure 2, under the normal situation, the controller first issues the command $c_{1}$ at the system state $s_{1}$, and then issues the command $c_{3}$. If $c_{3}$ is disaggregated before $c_{1}$ is disaggregated, the false command sequence occurs.

**Figure 4:**
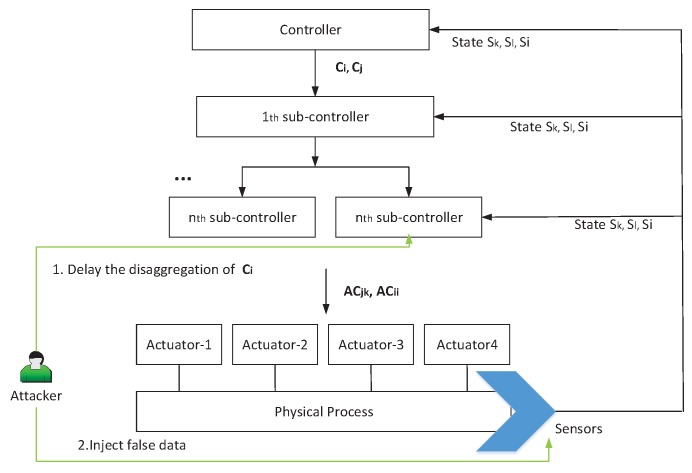
Attack model based on false command sequence mode.

As shown in Figure 4, the attack model (FCS) is described as follows:

• Information collection

Attackers first find command sequence $<C\left(t-d_{t}\right),C\left(t\right)>$ satisfying Equation (6).

(6) $$\left\{\begin{array}{c} c_{i}\in C\left(t-d_{t}\right),\\ c_{j}\in C\left(t\right).\\ \;<s_{k},c_{i},AC_{ik}>\in Re,\\ \;<s_{i},c_{i},AC_{ii}>\in Re,\\ s_{l}=A\times s_{k}+B\times AC\left(t-d_{t}\right),\\ s_{i}=A\times s_{l}+B\times AC\left(t\right),\\ s_{h}=A\times s_{k}+B\times \left(AC\left(t-d_{t}\right)-AC_{ik}\right),\\ s_{n}=A\times s_{h}+B\times AC\left(t\right),\\ s_{m}=A\times s_{n}+B\times \left(AC_{ii}\right),\\ s_{m}\in Fs. \end{array}\right.$$

• Time-delay attack

Attackers manipulate the sub-controllers to delay the disaggregation of $c_{i}$. The disaggregation of $c_{i}$ begins after the sub-commands from the disaggregation of $c_{j}$ are executed.

• False data injection

Commands $C(t-d_{t})$ are issued at state $s_{k}$. After sub-commands $AC\left(t-d_{t}\right)-AC_{ik}$ from the disaggregation of commands $C\left(t-d_{t}\right)-c_{i}$ are executed, the real physical system state is changed from $s_{k}$ to $s_{h}$. Because the current state is not $s_{l}$, the controller does not issue the commands C(t). Thus, attackers inject false data into sensors to induce false state estimation. The controller issues the commands C(t) when it obtains the false state $s_{l}$. After sub-commands AC(t) from the disaggregation of C(t) are executed, the state becomes $s_{n}$. To enable command $c_{i}$ to be disaggregated, attackers again inject false data to tell the controller and sub-controllers that the current state is $s_{i}$. When the sub-commands $AC_{ii}$ is executed, the real state is changed from $s_{n}$ to $s_{m}$ and a fault will occur. Attackers can enhance attack effect by avoiding anomaly discovery. To achieve this target, fault data can be continuously injected into controllers and detectors to tell them that the current state is $s_{i}$.

## 4. Detection Framework Based on Correlations among Two-Tier Command Sequences

WCIA, WCOA, and FCS change executed commands during the process of disaggregation, meanwhile, inject false data to confuse detectors. The existing detection methods in Section 2, such as false data evaluation [14] and event correlation based method that collects commands from the central controller [28], can not discover anomalies caused by these attacks. To fill the gap, we propose a novel and effective detection framework to identify attacks based on WCIA, WCOA, and FCS. We first describe the structure of the detection framework. Second, we examine how to mine correlations and use these correlations to identify anomalies caused by command disaggregation attacks.

### 4.1. Detection Framework

**Figure 5:**
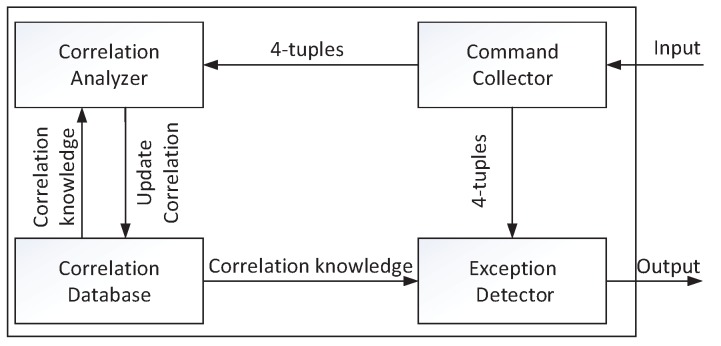
The structure of detection framework.

The detection framework is in charge of collecting command sequences, mining correlations, and identifying anomalies. As shown in Figure 5, the framework is comprised of command collector, correlation analyzer, correlation database, and exception detector. The functions of the four components are described below.

• Command Collector

Command collector is responsible for collecting commands from IIoTs. Command collector gets commands from two sites, as shown in Figure 1, including commands from the central controller and sub-commands from all actuator inputs. Every time a command collector receives a four-tuple $<C\left(k\right),k,AC\left(k\right),t_{AC(k)}>$, where $t_{AC(k)}=\left\{t_{AC(k)}\left(1\right),\ldots ,t_{AC(k)}\left(N\right)\right\}$, and $t_{AC(k)}\left(i\right)$ means the time when sub-command AC(i,k)∈AC(k) is executed by the *i*th actuator. Data is then transferred to two other components, namely, correlation analyzer and exception detector.

• Correlation Analyzer

Correlation analyzer tries to discover whether correlations exist among commands and sub-commands. Correlation analyzer mines correlations by using the recently collected history data. Once in a while the analyzer will update the correlations in correlation database. We will discuss which correlations and how they are mined in the next subsection.

• Correlation Database

Correlation information is stored in the correlation database. Correlation information includes discovered correlations and the time and number of occurrences of commands and sub-commands. Correlation database provides the corresponding information when the correlation analyzer or exception detector requires.

• Exception Detector

Exception detector examines anomalies of the input four-tuple based on correlation information. The exception detector directly utilizes correlations in database, instead of waiting for knowledge from the correlation analyzer, to identify anomalies. Therefore, the time that the detector spends in identifying anomalies is not related to correlation mining. The detector can provide the real-time result when a 4-tuple is input.

### 4.2. Correlation Mining and Exception Detection

We mainly mine two kinds of correlations including correlations between a command and sub-commands, and correlations between executed sub-commands.

#### 4.2.1. Correlation between a Command and Sub-Commands

If executed sub-command $ac_{ij}\left(k\right)$ can be obtained by the disaggregation of command $c_{l}$, there exists a correlation between command $c_{l}$ and sub-command $ac_{ij}\left(k\right)$, denoting as $<1,c_{l},ac_{ij}\left(k\right)>$. From an input four-tuple, we can not easily judge which command is correlated with an executed sub-command because multiple commands may be issued simultaneously from the central controller. We use greedy rules to mine this kind of correlation by analyzing a large number of four-tuples. We first define one parameter:

**Latter support ratio $P_{ac_{ij}\left(k\right)}\left(c_{l},ac_{ij}\left(k\right)\right)$:** denotes the ratio of the number of occurrences that $ac_{ij}\left(k\right)$ is executed when command $c_{l}$ is disaggregated, to the number of occurrences that command $ac_{ij}\left(k\right)$ is executed. It can be computed as

$$\frac{N(c_{l},ac_{ij}\left(k\right))}{N_{ac_{ij}\left(k\right)}}$$

where $N(c_{l},ac_{ij}\left(k\right))$ denotes the number of occurrences that $c_{l}$ is issued from the controller and $ac_{ij}\left(k\right)$ is executed by the actuator in an effective time interval $T_{interval}$. $N_{ac_{ij}\left(k\right)}$ means the number of occurrences that sub-command $ac_{ij}\left(k\right)$ is executed by the *k*th actuator. The value of $T_{interval}$ depends on the characters of physical system and transmission delay.

**Figure 6:**
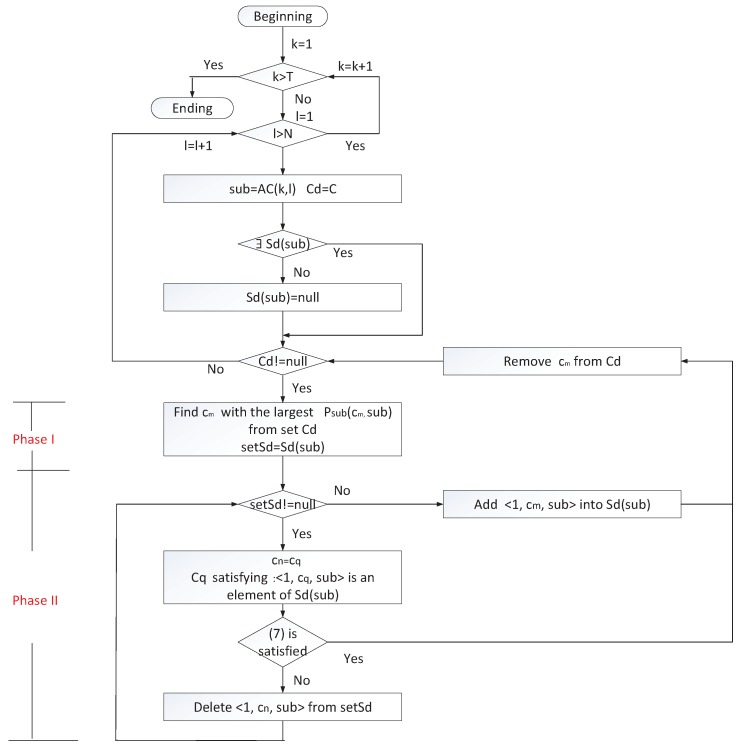
The flowchart of correlation mining between a command and a sub-command.

At the beginning of correlation mining, there exist many 4-tuples $\left\{<C\left(1\right),1,AC\left(1\right),t_{AC(1)}>,\ldots ,<C\left(k\right),k,AC\left(k\right),t_{AC(k)}>,\ldots ,<C\left(T\right),T,AC\left(T\right),t_{AC(T)}>\right\}$. The latter support ratio between any executed sub-command $AC\left(k,l\right)=ac_{ij}\left(l\right)$ and any command $c_{i}\in C$ is computed by analyzing these 4-tuples. For any sub-command $ac_{ij}\left(l\right)$, the process of mining which commands are correlated with the sub-command can be divided into two phases including verified correlation selection and correlation validation. The flowchart is shown in Figure 6 and the details are described as follows:

**Phase I: verified correlation selection.** In this phase, the correlation analyzer only needs to find a command $c_{m}$ satisfying

$$\max_{c_{m}\in Cd}P_{ac_{ij}\left(l\right)}\left(c_{m},ac_{ij}\left(l\right)\right)$$

where Cd is a set of commands and it is equal to *C* when correlation mining between sub-command $ac_{ij}\left(l\right)$ and commands begins.

**Phase II: correlation validation.** In the second phase, the correlation analyzer judges whether there exists a correlation between $c_{m}$ and $ac_{ij}\left(l\right)$.

We use $Sd(ac_{ij}\left(l\right))$ to denote the set that comprises correlations related to $ac_{ij}\left(l\right)$ that have been validated. If $c_{m}$ does not satisfy Equation (7), the correlation exists and we add the correlation $<\;1,c_{m},ac_{ij}\left(l\right)>$ into set $Sd(ac_{ij}\left(l\right))$. If $c_{m}$ satisfies Equation (7), the correlation does not exist. After that, $c_{m}$ is removed from set Cd.

(7) $$\left\{\begin{array}{c} T\left(c_{m},ac_{ij}\left(l\right)\right)⫋T\left(c_{n},ac_{ij}\left(l\right)\right),\\ <1,c_{n},ac_{ij}\left(l\right)>\in Sd\left(ac_{ij}\left(l\right)\right). \end{array}\right.$$

where $T(c_{m},ac_{ij}\left(l\right))$ is the set containing all time intervals from the time of issuing command $c_{m}$ to the time of executing $ac_{ij}\left(l\right)$ in history data, for example, $\left[k,t_{AC(k)}\left(l\right)\right]$ is an element of $T(c_{m},ac_{ij}\left(l\right))$.

The two phases are executed repetitively until set Cd is null.

#### 4.2.2. Correlation among Executed Sub-Commands

If Equation (8) is satisfied for executed sub-commands $ac_{mn}\left(i\right)$ and $ac_{pq}\left(j\right)$ that are correlated to command $c_{l}$, a correlation exists between $ac_{mn}\left(i\right)$ and $ac_{pq}\left(j\right)$, denoted as $<2,c_{l},ac_{mn}\left(i\right),ac_{pq}\left(j\right),\theta^{*}>$. $\theta^{*}$ means $\theta (c_{l},ac_{mn}\left(i\right),ac_{pq}\left(j\right))$. This kind of correlations denotes that there exists a linear relationship between the number of occurrences of two sub-commands.

(8) $$\left\{\begin{array}{c} \psi \left(k\right)={\left[-y\left(k-1\right),\ldots ,-y\left(k-p_{n}\right),x\left(k\right),\ldots x\left(k-p_{m}\right)\right]}^{T},\\ {\|y\left(k\right)-\psi \left(k\right)}^{T}\theta \left(c_{l},ac_{mn}\left(i\right),ac_{pq}\left(j\right)\right)\|<\varepsilon ,\\ \theta \left(c_{l},ac_{mn}\left(i\right),ac_{pq}\left(j\right)\right)={\left[a_{1},\ldots ,a_{p_{n}},b_{0},\ldots b_{p_{m}}\right]}^{T}, \end{array}\right.$$

where y(*k*) and x(*k*) indicate the number of occurrences that $ac_{mn}\left(i\right)$ is executed by the *i*th actuator and the number of occurrences that $ac_{pq}\left(j\right)$ is executed by the $j_{th}$ actuator when command $c_{l}$ is issued at its *k*th outflow. ε is the error threshold. $p_{n}$, $p_{m}$, and ε are input parameters and are obtained based on characters of system and data analysis.

**Figure 7:**
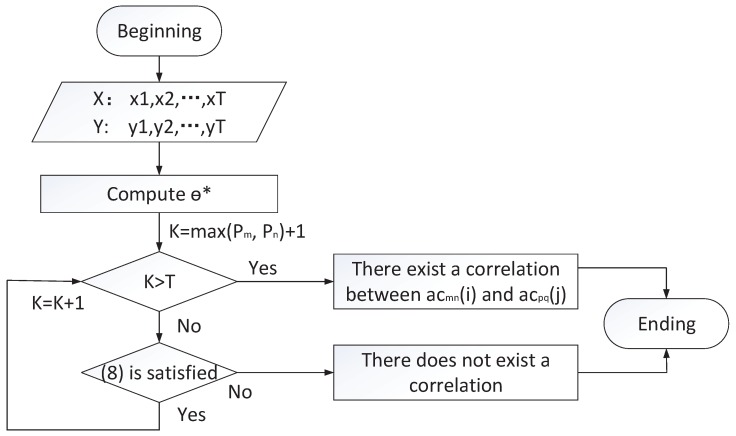
The flowchart of correlation mining between two sub-commands.

The flowchart of correlation mining among sub-commands is given in Figure 7. The key procedure is to compute $\theta^{*}$, which is elaborated as

The correlation analyzer can obtain $O_{N}=\left\{x\left(1\right),y\left(1\right),\ldots ,x\left(N\right),y\left(N\right)\right\}$ from the correlation database. $\theta^{*}$ is a constant vector and can be computed in Equation (9) by applying the least squares method to minimize estimation error $E_{N}\left(\theta ,O_{N}\right)$.

(9) $$\left\{\begin{array}{c} E_{N}\left(\theta^{*},O_{N}\right)=\frac{1}{N}\sum_{t=1}^{N}\left(y\left(t\right)-\psi {\left(t\right)}^{T}\theta^{*}\right),\\ \theta^{*}={\left[\sum_{t=1}^{N}\psi \left(t\right)\psi {\left(t\right)}^{T}\right]}^{-1}\sum_{t=1}^{N}\psi \left(t\right)y\left(t\right). \end{array}\right.$$

After computing $\theta^{*}$, we will validate whether (8) is satisfied for any k $\in [max\left(p_{m},p_{n}\right)+1,N]$. When (8) is satisfied for any k, the correlation exists. Otherwise, there does not exist a correlation between $ac_{mn}\left(i\right)$ and $ac_{pq}\left(j\right)$. Correlations and history data should be updated due to the degradation of system performance and changes in behaviors [15]. At the beginning of update, existing correlations will be directly removed from the database and new correlations are computed based on the mentioned process.

Lastly, we introduce the detection process of the exception detector.

Exception detector identifies anomalies based on broken correlations. For a sub-command $ac_{mn}\left(h\right)\in AC\left(k\right)$ from the input four-tuple $\left\{C\left(k\right),k,AC\left(k\right),t_{AC(k)}\right\}$, if we can not find a command $c_{i}\in C\left(k\right)$ to ensure that correlation $<1,c_{i},ac_{mn}\left(h\right)>$ exists in the database, then an alarm will be issued. For any correlation $<2,c_{l},ac_{mn}\left(i\right),ac_{pq}\left(j\right),\theta^{*}>$, if $c_{i}=c_{l}$ ($c_{i}\in C\left(k\right)$), the exception detector will verify whether the correlation is broken under the new command $c_{i}$ and sub-command AC(k). If an existing correlation is broken, an alarm is issued .

## 5. Case Study

In this section, we investigate two cases about tank system and energy trading system in the smart grid to illustrate the impact of attacks and the effectiveness of our detection framework.

### 5.1. Scenarios

#### 5.1.1. Scenario 1:3-Tank System

**Figure 8:**
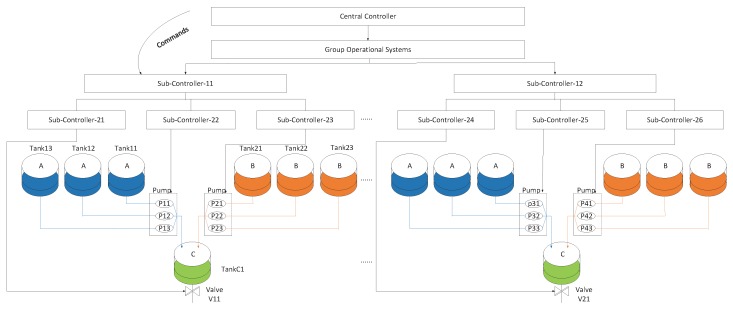
The structure of a tank system.

A tank system [16,32] with 100 sub-tank systems of the same liquid is utilized in this case. Figure 8 demonstrates the structure of tank system. The factory produces liquid C by the neutralization process of ingredient A and ingredient B. The ratio of ingredient A to ingredient B is 1. Error within 10% is allowed, and 1 mL A and 1 mL B can neutralize 2 mL liquid C. Ingredient A and ingredient B flow out from their tanks by 3 mL/second. Liquid C flows out from its tank by 6 mL/second. Every sub-system that can produce liquid C is composed of three tanks with ingredient A, three tanks with ingredient B, and one tank used to neutralize liquid C, six pumps used to output ingredient A or ingredient B, one valve used to output liquid C. When the central controller issues a command, Group Operational Systems will issue the same command to its all next-tier sub-controller.

We only describe a sub-system to illustrate the control process. Table 1 describes the 14 executed sub-commands and 7 sensors. Sensors S11, S12, and S13 measure the amount of ingredient A in Tank11, Tank12, and Tank13, respectively. Sensors S21, S22, and S23 measure the amount of ingredient B in Tank21, Tank22, and Tank23, respectively. Sv1 measures the amount of liquid C in TankC1.

A plan of producing M×3×2×100 mL liquid C within T=3×M+240 s is provided to the central controller. The central controller will continuously issue commands including *turning on the pump that outputs ingredient A* at time 0 s (pao), *turning off the pump that outputs ingredient A* at time M s (pac), *turning on the pump that outputs ingredient B* at time M + 60 s (pbo), *turning off the pump that outputs ingredient B* at time 2×M+60 s (pbc), *opening the valve that outputs liquid C* at time 2×M+180 s (pvo), and *closing the valve that outputs liquid C* at time 3×M+240 s (pvc). The process is executed repetitively if users have a new order of goods. When the sub-controller will output M×3 mL liquid A for a sub-system, it will open the pump with the largest amount of ingredient A until the output is equal to M×3 mL. If the tank with the largest amount of ingredient A is insufficient, the sub-controller will open other pumps to produce ingredient A. Thus, the sub-controller can simultaneously open multiple pumps to output ingredient A. For example, when users will produce M×3×4×100 mL liquid C within 3×M+240 s, the sub-system should open two pumps of outputting ingredient A. If sub-controllers open multiple pumps and receive the command “turning off the pump”, they also issue sub-commands to turn off multiple pumps. The corresponding ingredient will be supplied when two or more tanks with ingredient A or ingredient B are empty. The initial volume of every tank with ingredient A or ingredient B is 60×6×3 mL.

The above neutralization process depicted is simulated in Java, where the central controller, actuators, and sub-controllers are designed as components by using Java Class. Every switch and sensor are seen as attributes of related actuators. When some attributes occur a change, the central controller issues new commands. Some executed sub-commands can cause the changes of the attributes. Different components communicate with each other by function call with parameters. The parameters include commands and feedback data. The central controller automatically keeps running and issues commands based on the users’ input and the designed control process. During the operation of the system, values of sensors, sub-commands, and commands are written into different files per unit time. Moreover, every sub-controller component provides an interface for users. When users call the interface and input parameters, sub-controllers have been compromised and commands and feedback data can be modified.

#### 5.1.2. Scenario 2: Energy Trading System in the Smart Grid

With the increasing proliferation of new energy, many users can become suppliers who sell energy to other users called consumers. Every supplier has an energy storage system that stores extra energy. When consumers need to buy energy, energy is routed to these consumers from suppliers based on energy routing schemes.

**Figure 9:**
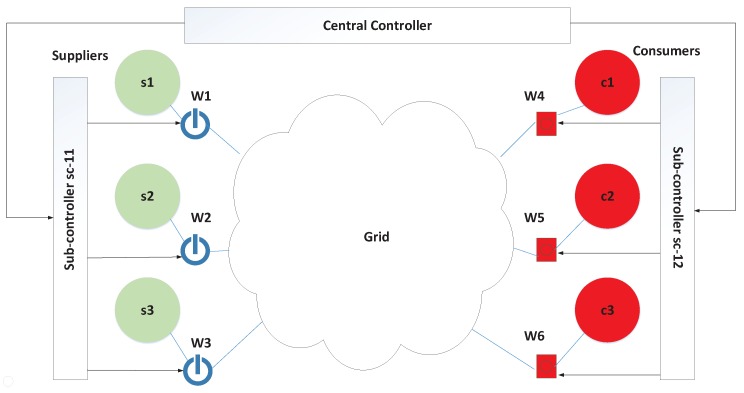
The model of energy trading system in the smart grid.

A simplified model of energy trading system in the smart grid [33,34] is shown in Figure 9, where there are 3 suppliers and 3 consumers. The central controller receives sensory data from consumers and suppliers and sends commands to control switches that are responsible for outputting or inputting energy. Sensory data from the consumers describes how much energy has been input and data from the suppliers depicts how much energy can be outputted. When a switch is turned on, energy can be input or be outputted by 500 w/s. When the output of energy is larger than the demands of consumers, extra energy will be wasted. When supplied energy can not satisfy demands of consumers, some consumers have to turn off some appliances. If the amount of energy routed to a consumer is larger than his demands, extra cost needs to be paid. The control process needs to try to avoid the above three situations.

In the model, there are 12 sub-commands and 6 sensors, which are shown in Table 2. Sensors Ss1, Ss2, and Ss3 measure the amount of energy that can be provided by suppliers s1, s2, and s3, respectively. Sensors Sc1, Sc2, and Sc3 measure the amount of energy that has been bought by consumers c1, c2, and c3, respectively.

At the beginning of every circle, consumers sent their demands K×500 w and suppliers send the amount of their energy to the central controller. The central controller will continuously issue commands including *turning on the switch that outputs energy* at time 0 s (Ooute), *turning on the switch that inputs energy* at time 0 s (Opute), *turning off the switch that outputs energy* at time *K* s (Coute), *turning off the switch that inputs energy* at time K+10 s (Opute). When the suppliers will output *K* w energy, it will turn on the switch with the largest amount of energy until the output is equal to *K* w. If multiple users request power, the sub-controller will turn on other switches to output energy. The initial volume of every storage system is 60×6×500 w. Energy will be compensated when two or more suppliers can not supply enough energy. The described model with the trading process is also simulated in Java and the details of implementation are similar to scenario 1.

### 5.2. Attack Cases

**Figure 10:**
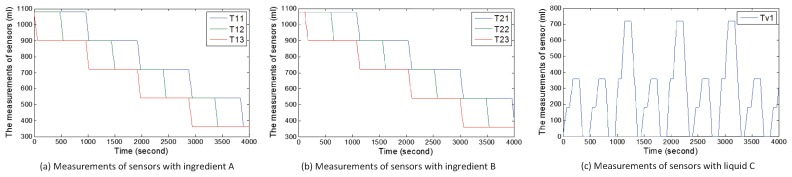
Measurements from sensors under the normal situation of scenario 1.

**Figure 11:**
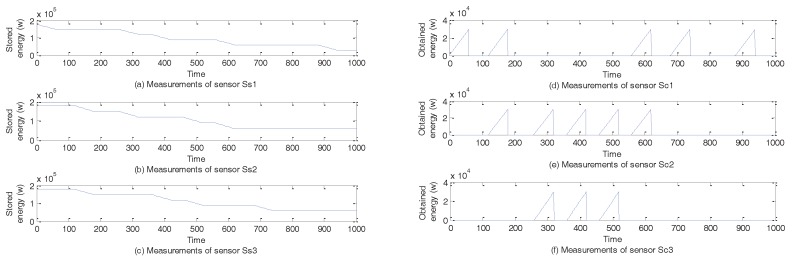
Measurements from sensors under the normal situation of scenario 2.

In this subsection, we introduce six attack cases based on WCOA, WCIA and FCS. Under normal circumstances of scenario 1, users randomly receive orders of goods including 60×3×2×100 mL, 60×3×4×100 mL, and 60×3×6×100 mL. We show the normal measurements of sensors with the change of time under random orders of goods in Figure 10. Figure 10a,b show the measurements of sensors about ingredients A and B. Figure 10c shows the amount of liquid C. When the value in TankC1 reaches the highest point in a cycle, the ratio of ingredient A to ingredient B is 1. Hence, liquid C can be obtained. Under normal circumstances of scenario 2, consumers randomly receive orders of energy including 60×500 w (one consumer) and 60×2×500 w (two consumers). We also show the normal measurements of sensors with the change of time under random orders of energy in Figure 11. Figure 11a–c show the amount of energy in storage systems of three suppliers. Figure 11d–f show the amount of energy obtained by three consumers in every circle.

Six attack cases are described as follows:

**Attack case 1 in scenario 1:** When the controller issues command pao, malicious entities launch attacks based on WCIA to turn on pump p13 by telling sub-controller sc-22 that Tank13 has the most ingredient A.

**Attack case 2 in scenario 1:** At t=960 s, the controller issues the command pao and malicious entities launch attacks based on WCOA by manipulating the sub-controller sc-11 and modifying state feedback to sc-22 and sc-23. Operation “turning on the pump p11” is replaced by the operation “turning on the pump p21”.

**Attack case 3 in scenario 1:** At t=0 s, the controller issues command pao and attackers launch attacks based on FCS. Malicious entities first manipulate sub-controller sc-22 not to disaggregate command pao. Command pao is disaggregated after command pac is disaggregated at t=60 s.

**Attack case 4 in scenario 2:** When the controller issues command Ooute, malicious entities launch attacks based on WCIA to turn on w1 by telling sub-controller sc-11 that supplier s1 has the most energy.

**Attack case 5 in scenario 2:** At t=0 s, the controller issues the command Ooute and malicious entities launch attacks based on WCOA by manipulating the sub-controller sc-11 and modifying state feedback to sc-11. Operation “turning on the switch w1” is replaced by the operation “turning off the switch w2”.

**Attack case 6 in scenario 2:** At t=0 s, the controller issues command Ooute and attackers launch attacks based on FCS. Malicious entities first manipulate sub-controller sc-11 not to disaggregate command Ooute. Command Ooute is disaggregated after command Coute is disaggregated at t=60 s.

During the above attack processes, attackers also modify data of sensors to confuse the central controller and detectors, thereby resulting in sensory data same to those in Figure 10 and Figure 11.

### 5.3. Attack Impact

#### 5.3.1. Case 1

**Figure 12:**
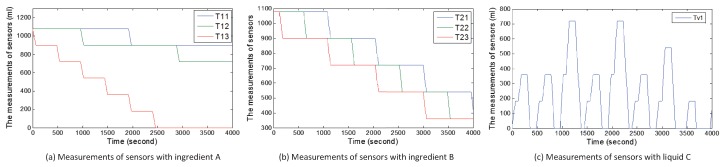
Measurements from sensors under attack case 1.

Figure 12 demonstrates the real measurements of sensors under attack case 1. Real measurements refer to the real values of sensors, which may be different from received sensory data by the central controller or state estimator. Compared with Figure 10, ingredient B normally flows into TankC1. However, the change in the amount of ingredient A is abnormal since t=480 s. From the beginning of the second circle, Tank13 always outputs ingredient A until the tank is empty. Before Tank13 is empty, the ratio of ingredient A to ingredient B in TankC1 is 1 and the factory can produce liquid C. When Tank13 is empty, the sub-controller still turns on pump p13 and outputs ingredient A from Tank13, which leads to a false ratio and fails to produce liquid C. Figure 12c demonstrates the above process. The ratio is false and liquid C can not be obtained in the seventh circle.

#### 5.3.2. Case 2

**Figure 13:**
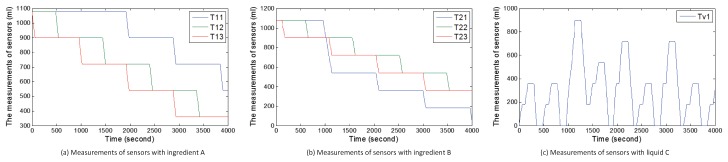
Measurements from sensors under attack case 2.

Figure 13 describes the real measurements of sensors under attack case 2. Unlike in Figure 10, ingredient A in Tank12 and Tank13 is normal. From t=960 s, ingredient A should be outputted from Tank11 and Tank13. However, ingredient A is only outputted from Tank13 in Figure 13a. In the third circle (from t=960 s to t=1440 s), 180 mL ingredient A flows into Tank13. In Figure 13b, 540 mL ingredient B flows into TankC1 from Tank21 from t=960 s to t=1440 s and 180 mL of ingredient B flows into TankC1 from Tank23. The ratio of ingredient A to ingredient B is not 1 and liquid C can not be produced. At the beginning of the fourth circle, the TankC1 is not empty, but users still obtain the wrong product.

#### 5.3.3. Case 3

**Figure 14:**
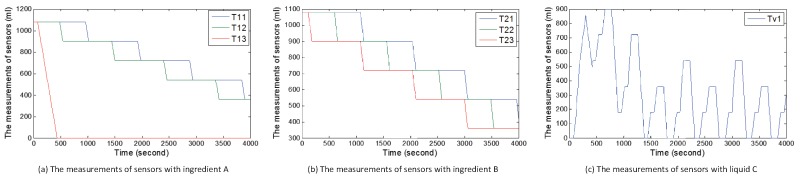
Measurements from sensors under attack case 3.

Figure 14 describes the real values of sensors under attack case 3. The change in the amount of ingredient B is normal in Figure 14b. Unlike in Figure 10a, Figure 14a shows that the change in the amount of ingredient A in Tank13 is abnormal. When turning off pump p13 occurs before turning on pump p13, the ingredient A will be outputted continuously from Tank13. At t=480, users can not obtain the liquid C because of false ratio. At the beginning of the second circle, the liquid in TankC1 still exists and liquid C is still not obtained. It also fails to obtain liquid C at the third circle, the fifth circle and the sixth circle because the amount of ingredient A in Tank13 is zero.

#### 5.3.4. Case 4–Case 6

**Figure 15:**
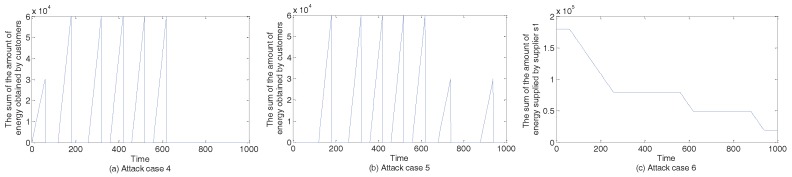
Impact of attack case 4, attack case 5, and attack case 6.

Figure 15 describes the sum of the amount of supplied energy or the sum of the amount of energy obtained by consumers under attack cases 4–6. In Figure 15a, we show the sum of the amount of energy obtained by consumers under attack case 4. Compared with the situation in Figure 11d–f, we can find that at the 7th circle and the 8th circle, consumers can not buy energy. That is because when the central controller turns on the switch w1 to output energy, energy in the storage system of supplier s1 is zero because of attacks. In Figure 15b, we also show the sum of the amount of energy obtained by consumers under attack case 5. Compared with the situation in Figure 11d–f, we can find that in the first circle, consumer c1 can not buy energy. That is because when attacks occur, the switch s1 is not turned on and energy can not be outputted. In Figure 15c, the sum of the amount of energy supplied by supplier s1 is shown. We can clearly see that supplier s1 does not output energy at the first circle, which enables consumers to turn off some appliances. Moreover, supplier 1 loses a large amount of energy in the interval from time t=60 to time t=260.

The above six cases demonstrate that command disaggregation attacks can lead to disruptions of physical process and create great impact.

### 5.4. Effectiveness of Our Detection Framework

We employed java to implement the detection framework described in Section 4, where every component is described as a Java class and we use MySQL Database software as the correlation database. Different components use functions to exchange information with the database. In each component, we add functions to implement the corresponding operations. We analyze the data from tank system in Figure 8 and energy trading system in Figure 9. The data is collected from the files that are written by tank system and energy trading system. The information comprises sensory data from sensors, commands from the central controller, and executed commands from the actuators. We set $T_{interval}=60$ s. Data is collected from t=0 s to $t=3\times 10^{6}$ s under random orders of goods and energy purchase.

**Figure 16:**
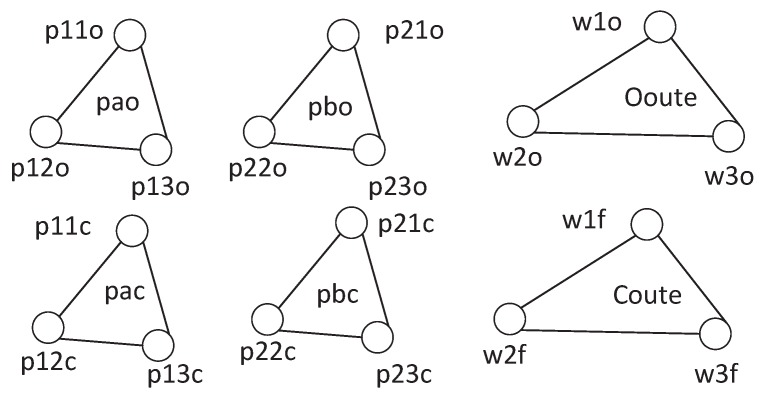
Correlations between executed sub-commands. A link denotes two correlations between two nodes.

We check whether the proposed detection framework can effectively identify six attack cases. Two kinds of correlations are obtained by analyzing data. In Table 3, the correlations between a command and executed sub-commands are described. 14 correlations in scenario 1 and 12 correlations in scenario 2 are discovered. To mine correlations between sub-commands, we set parameters $p_{n}=0$, $p_{m}=2$, and ε=1. We can obtain 24 correlations in scenario 1 and 12 correlations in scenario 2 as shown in Figure 16. Any link between two sub-commands can denote two existing correlations between two sub-commands. For example, the correlations between two sub-commands, p11o and p13o, are described as

$$\begin{array}{c} F(pao,p11o,t)=0.75F(pao,p13o,t)-1+0.25F(pao,p13o,t-1)\\ F(pao,p13o,t)=0.65F(pao,p11o,t)+1+0.35F(pao,p11o,t-1) \end{array}$$

where F(pao,p11o,t) denotes the number of occurrences that p11o is executed when pao is disaggregated at its $t_{th}$ outflow, and F(pao,p13o,t) indicates the number of occurrences that p13o is executed when pao is disaggregated at its $t_{th}$ outflow.

Table 4 displays the results of detection based on two types of correlations under six attack cases. Results show that when attackers launch a command disaggregation attack based on WCIA in scenario 1 at t=480 s, the correlation between sub-commands will be broken and the alarms are instantly shown. When an attack based on WCOA at t=960 s is launched in scenario 1, there does not exist a correlation between command pao and sub-command p22o in the database and defenders can achieve the alarms instantly. When an attack based on FCS in scenario 1 occurs at t=0 s, there does not exist a correlation between command pac and sub-command p11o. An alarm is issued until the disaggregation of the next command occurs at t=60 s. When attackers launch a command disaggregation attack based on WCIA in scenario 2 at t=0 s, the correlation between sub-commands will be broken and the alarms are instantly shown. When an attack based on WCOA at t=0 s is launched in scenario 2, there does not exist a correlation between command Ooute and sub-command w2f in the database and defenders can achieve the alarms instantly. When an attack based on FCS in scenario 2 occurs at t=0 s, there does not exist a correlation between command Coute and sub-command w1f. An alarm is issued until the disaggregation of the next command occurs at t=60 s. These alarms can be obtained when false sub-commands are executed, and occur before disruptions of physical process. During the process, our detection framework does not issue false alarms, which demonstrates that our methods of correlation mining are effective.

We also implement two other detection methods in [14,35] to detect six attack cases. Because false feedback data is injected into the state estimator, the detection method in [14] cannot identify six attack cases. The detection method in [35] also does not show any exception under the six attack cases because detectors use commands from the central controller.

To better illustrate the performance of the proposed detection framework, we randomly launch attacks based on WCIA, WCOA, and FCA in scenario 1 and scenario 2. Every type of attack is launched many times at the different time. We find that attacks based on FCA and WCOA can be identified with 100% accuracy in two scenarios. Attacks based on WCIA in scenario 1 can be identified with 95% accuracy because some elaborately constructed attacks enable the correlation between two sub-commands not to be broken. An example will be described in Section 6. 47.5 % attacks based on WCIA in scenario 2 can be identified. It is lower than the accuracy in scenario 1 because there does not exist a correlation among sub-commands w4o,w5o,w6o,w4f,w5f, and w6f. When correlations can not be mined among sub-commands, attacks based on WCIA may not be detected. For the detection of the above attacks, the detection framework does not issue any false alarm.

The experiments demonstrate that the detection framework can effectively identify many command disaggregation attacks, and can find anomalies before disruptions of physical process occur.

## 6. Discussion of Detection Framework Enhancement

This section discusses further improvement measures for the defects of our detection framework.

**Difficulties of correlation mining.** A large number of linear relationships exist among data of complex IIoTs [36], however, the relationship between two sub-commands may be nonlinear, which can increase the difficulty of identifying command disaggregation attacks. Thus, the detection framework can utilize other methods, such as information theory [35] to identify nonlinear relationships, which can be decided by defenders.

**The futility of detecting elaborately constructed attack sequences.** Experiments in Section 5 have shown the effectiveness of the detection framework. While the priest climbs a post, the devil climbs ten. Hence, if attackers launch attacks based on WCIA without breaking correlations between sub-commands, the proposed detection framework may not issue an alarm. For example, when the system in Figure 8 attempts to output ingredient A by merely opening a pump, attackers can open two pumps to output additional ingredient A, which can conduct false ratio of ingredient A to ingredient B. When the normal sub-command sequence is {<p11o,t=0>,<p12o,t=480>,<p13o,t=960>} and attackers continuously manipulate the sub-controllers to issue command sequence {<p11o,t=0>,<p12o,t=0>,<p13o,t=480>,<p11o,t=480>,<p12o,t=960>,<p13o,t=960>}, correlations among sub-commands are not broken. To cope with the tricky attack, defenders can improve the performance by mining correlations among more types of data, e.g., mining the linear relationship between the number of opening pumps that output ingredient A and the number of opening pumps that produce ingredient B. The numbers are equal in the normal situation, but the relationship is broken under the above attacks and an alarm can be issued.

## 7. Conclusions

In this study, we focus on the command disaggregation attack and its detection method. We describe three attack models to implement command disaggregation attacks in two kinds of modes. The examples of the tank system and energy trading system demonstrate that command disaggregation attacks in two modes can cause severe damage to physical process and an effective detection method is necessary. We also provide a novel framework to detect command disaggregation attacks. The framework utilizes the correlations between commands and sub-commands to identify anomalies. The two cases demonstrate that our detection framework can identify undetected command disaggregation attacks by the existing detection methods with high accuracy if there exist corresponding correlations among commands and sub-commands. Besides that, our method can identify anomalies before a fault occurs. In future, we will strengthen the detection framework to detect command disaggregation attacks in more complex IIoTs.

## Figures and Tables

**Table 1:** Description of data in the tank system.

Command/Time Series	Description
P11o/P11f	Switch on/off Pump P11
P12o/P12f	Switch on/off Pump P12
P13o/P13f	Switch on/off Pump P13
P21o/P21f	Switch on/off Pump P21
P22o/P22f	Switch on/off Pump P22
P23o/P23f	Switch on/off Pump P23
V11o/V11c	Open/Close Valve V11
T11	Measurements of Sensor S11
T12	Measurements of Sensor S12
T13	Measurements of Sensor S13
T21	Measurements of Sensor S21
T22	Measurements of Sensor S21
T23	Measurements of Sensor S23
Tv1	Measurements of Sensor Sv1

**Table 2:** Description of data in the energy trading system.

Command/Time Series	Description
w1o/w1f	Turn on/off switch w1
w2o/w2f	Turn on/off switch w2
w3o/w3f	Turn on/off switch w3
w4o/w4f	Turn on/off switch w4
w5o/w5f	Turn on/off switch w5
w6o/w6f	Turn on/off switch w6
T11	Measurements of Sensor Ss1
T12	Measurements of Sensor Ss2
T13	Measurements of Sensor Ss3
T21	Measurements of Sensor Sc1
T22	Measurements of Sensor Sc1
T23	Measurements of Sensor Sc3

**Table 3:** Correlations between commands and executed sub-commands.

Command	Correlation	Scenario
pao	<pao,p11o>, <pao,p12o>, <pao,p13o>	1
pbo	<pbo,p21o>, <pbo,p22o>, <pbo,p23o>	1
pac	<pac,p11c>, <pac,p12c>, <pac,p13c>	1
pbc	<pbc,p21c>, <pbc,p22c>, <pbc,p23c>	1
pvo	<pvo,v11o>	1
pvc	<pvc,v11c>	1
Ooute	<Ooute,w1o>, <Ooute,w2o>, <Ooute,w3o>	2
Opute	<Opute,w4o>, <Opute,w5o>, <Opute,w6o>	2
Coute	<Coute,w1f>, <Coute,w2f>, <Coute,w3f>	2
Cpute	<Cpute,w4f>, <Cpute,w5f>, <Cpute,w6f>	2

**Table 4:** Broken correlations under attacks.

Attack Case	Alarm
1	<pao,p13o,p11o>, <pao,p13o,p12o>, <pao,p12o,p13o> at t=480 s
2	<pao,p22o> at *t* = 960 s
3	<pac,p11o> at *t* = 60 s
4	<Ooute,w1o,w2o>, <Ooute,w1o,w3o>, <Ooute,w2o,w1o> at t=0 s
5	<Ooute,w2f> att=0 s
6	<Coute,w1o> att=60 s

## References

[1] Fraga-Lamas P., Fernández-Caramés T.M., Castedo L. (2017). Towards the Internet of Smart Trains: A Review on Industrial IoT-Connected Railways. Sensors.

[2] Min B., Varadharajan V. Cascading Attacks Against Smart Grid Using Control Command Disaggregation and Services. Proceedings of the 31st Annual ACM Symposium on Applied Computing.

[3] Taft J.D. (2012). Control Command Disaggregation and Distribution within A Utility Grid. U.S. Patent.

[4] Remmersmann T., Schade U., Schlick C. Supervisory control of multi-robot systems by disaggregation and scheduling of quasi-natural language commands. Proceedings of the 2012 IEEE International Conference on Systems, Man, and Cybernetics (SMC).

[5] Zhao C., Topcu U., Low S.H. (2013). Optimal Load Control via Frequency Measurement and Neighborhood Area Communication. IEEE Trans. Power Syst..

[6] Hu J., Cao J., Guerrero J.M., Yong T., Yu J. (2017). Improving Frequency Stability Based on Distributed Control of Multiple Load Aggregators. IEEE Trans. Smart Grid.

[7] Sargolzaei A., Yen K., Abdelghani M. Delayed inputs attack on load frequency control in smart grid. Proceedings of the Innovative Smart Grid Technologies Conference (ISGT).

[8] Mitchell R., Chen I.R. (2016). Modeling and Analysis of Attacks and Counter Defense Mechanisms for Cyber Physical Systems. IEEE Trans. Reliab..

[9] Amini S., Mohsenian-Rad H., Pasqualetti F. Dynamic load altering attacks in smart grid. Proceedings of the 2015 IEEE Power Energy Society Innovative Smart Grid Technologies Conference (ISGT).

[10] Liu Y., Ning P., Reiter M.K. False Data Injection Attacks Against State Estimation in Electric Power Grids. In Proceedings of the 16th ACM Conference on Computer and Communications Security.

[11] Yi P., Zhu T., Zhang Q., Wu Y., Li J. A denial of service attack in advanced metering infrastructure network. Proceedings of the 2014 IEEE International Conference on Communications (ICC).

[12] Asri S., Pranggono B. (2015). Impact of Distributed Denial-of-Service Attack on Advanced Metering Infrastructure. Wirel. Pers. Commun..

[13] Gacia L.A., Brasser F., Cintuglu M.H., Sadeghi A.R. Hey, My Malware Knows Physics Attacking PLCs with Physical Model Aware Rootkit. Proceedings of the Network & Distributed System Security Symposium.

[14] Vu Q.D., Tan R., Yau D.K.Y. On applying fault detectors against false data injection attacks in cyber-physical control systems. Proceedings of the IEEE INFOCOM 2016—The 35th Annual IEEE International Conference on Computer Communications.

[15] Vuong T.P., Loukas G., Gan D., Bezemskij A. Decision tree-based detection of denial of service and command injection attacks on robotic vehicles. Proceedings of the 2015 IEEE International Workshop on Information Forensics and Security (WIFS).

[16] Li W., Xie L., Deng Z., Wang Z. (2016). False sequential logic attack on SCADA system and its physical impact analysis. Comput. Secur..

[17] Quarta D., Pogliani M., Polino M., Maggi F. An Experimental Security Analysis of an Industrial Robot Controller. Proceedings of the 2017 IEEE Symposium on Security and Privacy (SP).

[18] Tan R., Nguyen H.H., Foo E.Y.S., Dong X. Optimal False Data Injection Attack against Automatic Generation Control in Power Grids. Proceedings of the 2016 ACM/IEEE 7th International Conference on Cyber-Physical Systems (ICCPS).

[19] Li B., Lu R., Wang W., Choo K.K.R. (2017). Distributed host-based collaborative detection for false data injection attacks in smart grid cyber-physical system. J. Parallel Distrib. Comput..

[20] Wang J.K., Peng C. Analysis of Time Delay Attacks Against Power Grid Stability. Proceedings of the 2nd Workshop on Cyber-Physical Security and Resilience in Smart Grids.

[21] Guo Y., Ten C.W., Hu S., Weaver W.W. Modeling distributed denial of service attack in advanced metering infrastructure. Proceedings of the 2015 IEEE Power Energy Society Innovative Smart Grid Technologies Conference (ISGT).

[22] Hu F., Lu Y., Vasilakosb A.V., Hao Q., Ma R., Patila Y., Zhang T., Lua J., Lia X., Xiong N.N. (2016). Robust cyber physical systems: Concept, models, and implementation. Future Gener. Comput. Syst..

[23] Cheng W., Zhang K., Chen H., Jiang G. Ranking Causal Anomalies via Temporal and Dynamical Analysis on Vanishing Correlations. Proceedings of the 22nd ACM SIGKDD International Conference on Knowledge Discovery and Data Mining.

[24] Momtazpour M., Zhang J., Rahman S., Sharma R., Ramakrishnan N. Analyzing Invariants in Cyber-Physical Systems Using Latent Factor Regression. Proceedings of the 21th ACM SIGKDD International Conference on Knowledge Discovery and Data Mining.

[25] Luo C., Lou J.G., Lin Q., Fu Q., Ding R. Correlating Events with Time Series for Incident Diagnosis. Proceedings of the 20th ACM SIGKDD International Conference on Knowledge Discovery and Data Mining.

[26] Melnyk I., Banerjee A., Matthews B., Oza N. Semi-Markov Switching Vector Autoregressive Model-Based Anomaly Detection in Aviation Systems. Proceedings of the 22nd ACM SIGKDD International Conference on Knowledge Discovery and Data Mining.

[27] Wang J., Tu W., Hui L.C.K., Yiu S.M., Wang E.K. Detecting Time Synchronization Attacks in Cyber-Physical Systems with Machine Learning Techniques. Proceedings of the 2017 IEEE 37th International Conference on Distributed Computing Systems (ICDCS).

[28] Budalakoti S., Srivastava A.N., Otey M.E. (2009). Anomaly Detection and Diagnosis Algorithms for Discrete Symbol Sequences with Applications to Airline Safety. IEEE Trans. Syst. Man Cybern. Part C (Appl. Rev.).

[29] Lim H.K., Kim Y., Kim M.K. (2017). Failure Prediction Using Sequential Pattern Mining in the Wire Bonding Process. IEEE Trans. Semicond. Manuf..

[30] Ouaddah A., Elkalam A.A., Ouahman A.A. (2017). FairAccess: A new Blockchain-based access control framework for the Internet of Things. Secur. Commun. Netw..

[31] Liu B., Yu X.L., Chen S., Xu X., Zhu L. Blockchain Based Data Integrity Service Framework for IoT Data. Proceedings of the 2017 IEEE International Conference on Web Services (ICWS).

[32] Renganathan K., Bhaskar V. (2010). Observer based on-line fault diagnosis of continuous systems modeled as Petri nets. ISA Trans..

[33] Rahmani-andebili M., Shen H. Cooperative distributed energy scheduling for smart homes applying stochastic model predictive controla. Proceedings of the 2017 IEEE International Conference on Communications (ICC).

[34] Zhou Y., Ci S., Li H., Yang Y. A new framework for peer-to-peer energy sharing and coordination in the energy internet. Proceedings of the 2017 IEEE International Conference on Communications (ICC).

[35] Jiang M., Munawar M.A., Reidemeister T., Ward P.A.S. (2011). Efficient Fault Detection and Diagnosis in Complex Software Systems with Information-Theoretic Monitoring. IEEE Trans. Dependable Secur. Comput..

[36] Sharma A.B., Chen H., Ding M., Yoshihira K., Jiang G. Fault detection and localization in distributed systems using invariant relationships. Proceedings of the 2013 43rd Annual IEEE/IFIP International Conference on Dependable Systems and Networks (DSN).

